# Respiratory Evolution Facilitated the Origin of Pterosaur Flight and Aerial Gigantism

**DOI:** 10.1371/journal.pone.0004497

**Published:** 2009-02-18

**Authors:** Leon P. A. M. Claessens, Patrick M. O'Connor, David M. Unwin

**Affiliations:** 1 Department of Biology, College of the Holy Cross, Worcester, Massachusetts, United States of America; 2 Department of Biomedical Sciences, Ohio University College of Osteopathic Medicine, Athens, Ohio, United States of America; 3 Department of Museum Studies, University of Leicester, Leicester, United Kingdom; University of Chicago, United States of America

## Abstract

Pterosaurs, enigmatic extinct Mesozoic reptiles, were the first vertebrates to achieve true flapping flight. Various lines of evidence provide strong support for highly efficient wing design, control, and flight capabilities. However, little is known of the pulmonary system that powered flight in pterosaurs. We investigated the structure and function of the pterosaurian breathing apparatus through a broad scale comparative study of respiratory structure and function in living and extinct archosaurs, using computer-assisted tomographic (CT) scanning of pterosaur and bird skeletal remains, cineradiographic (X-ray film) studies of the skeletal breathing pump in extant birds and alligators, and study of skeletal structure in historic fossil specimens. In this report we present various lines of skeletal evidence that indicate that pterosaurs had a highly effective flow-through respiratory system, capable of sustaining powered flight, predating the appearance of an analogous breathing system in birds by approximately seventy million years. Convergent evolution of gigantism in several Cretaceous pterosaur lineages was made possible through body density reduction by expansion of the pulmonary air sac system throughout the trunk and the distal limb girdle skeleton, highlighting the importance of respiratory adaptations in pterosaur evolution, and the dramatic effect of the release of physical constraints on morphological diversification and evolutionary radiation.

## Introduction

Pterosaurs were the first vertebrates to evolve true flapping flight, a complex and physiologically demanding activity that required profound anatomical modifications, most notably of the forelimb [1]–[7], but which subsequently conferred great success in terms of clade longevity and diversity. Following a basal radiation in the Late Triassic, pterosaurs diversified into a wide variety of continental and marine ecosystems and remained successful aerial predators until the end of the Cretaceous, an interval of more than 150 million years [1], [2], [5], [7]. Efforts directed at understanding the history and biology of pterosaurs have long been hindered by their comparatively poor fossil record, attributable to a relative lack of preservation in lacustrine and fluvial sediments, and the nature of a skeleton composed of lightly built, hollow bones. Consequently, most pterosaur skeletons are highly compressed, with the fine anatomical details and three-dimensional spatial relationships of bones often distorted, obscured or lost.

**Figure 1:**
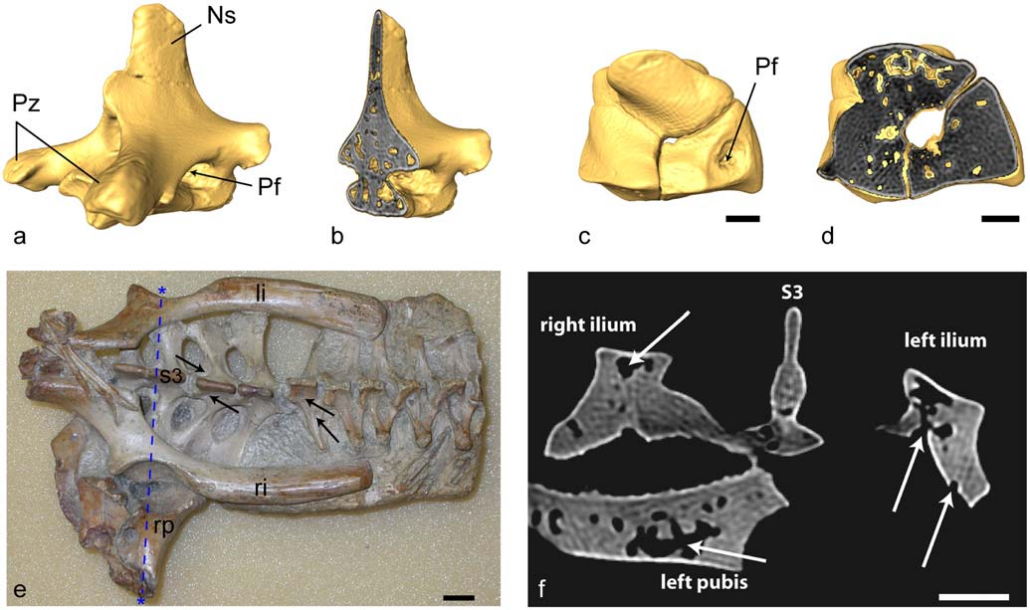
Micro-computed tomographic (CT) scans and photograph illustrating external pneumatic openings and typical pneumatic architecture in the ornithocheirid pterosaur *Anhanguera santanae* (AMNH 22555). Vertebral (a, b), carpal (c, d), and pelvic (e, f) elements are characterized by the presence of thin cortical bone and large internal cavities (b, d, f). a, b, Mid-cervical (6^th^) vertebra in oblique craniolateral (a) and cutaway oblique craniolateral (b) views. Vertebral height = 5 cm. c, d, Left distal syncarpal in proximal (c) and cutaway proximal (d) views. e, dorsal view of block with pelvic elements, sacral vertebrae, and posterior dorsal vertebrae. Black arrows indicate the location of pneumatic foramina on select vertebrae (e) and white arrows indicate both pneumatic foramina and internal pneumatic cavities on pelvic elements (f). Asterisks on (e) delineate the location of the transverse section (dashed blue line) shown in (f). f, Transverse CT scan transect through pelvic block, showing pneumaticity of the sacral neural spine, ilia and left pubis. Note the large pneumatic opening on the surface of the left ilium. Scale bar (c–f) = 1 cm. li, left ilium; Ns, neural spine; Pf, pneumatic foramen; Pz, prezygapophysis; ri, right ilium; rp, right pubis; s3, sacral vertebra 3.

Few studies have focused on pterosaurian respiration and information available in the literature is limited. Prior analyses are generally limited to isolated anatomical systems such as the prepubis [8], or to a small fraction of total taxonomic coverage such as derived pterodactyloids [9], [10]. Inferences generated thus far have implied a near-immobile ribcage associated with a pulmonary system similar to that of extant reptiles, thereby supposedly encumbering the clade with an ectotherm-like routine metabolic rate [9], [10], or present a more equivocal interpretation of affinity with either an avian or a crocodylian-like respiratory system [8]. Non-avian sauropsids exhibit a wide range of diversity in respiratory anatomy and performance [11]–[15]. Thus, recent evidence indicative of highly efficient flight capabilities [2], [6], [7], [16], [17] brings forward interesting questions regarding the structure and function of the respiratory system that powered the metabolic demands of pterosaurian flight. We investigated the pterosaurian breathing apparatus by utilizing recent developments in our understanding of the relationships between the skeletal and respiratory systems in extant tetrapods, especially birds and crocodylians [18]–[25], as a framework for interpreting ventilatory potential in pterosaurs. This study focused on examples of both basal (*Eudimorphodon* and *Rhamphorhynchus*) and derived pterosaurs (*Pteranodon* and *Anhanguera*) in which trunk structure has been well preserved. A dataset generated by computer-assisted tomographic (CT) scanning of a near-complete, three-dimensionally preserved skeleton of the Lower Cretaceous ornithocheirid *Anhanguera* (Fig. 1a–f) served as a comparative reference for a survey examining the distribution of postcranial pneumaticity in pterosaurs. Cineradiographic (X-ray film) studies of the skeletal kinematics of lung ventilation in alligators and birds provided a structural framework for our reconstruction of the pterosaurian breathing pump [26], [27].

## Results and Discussion

### The skeletal breathing pump

**Figure 2:**
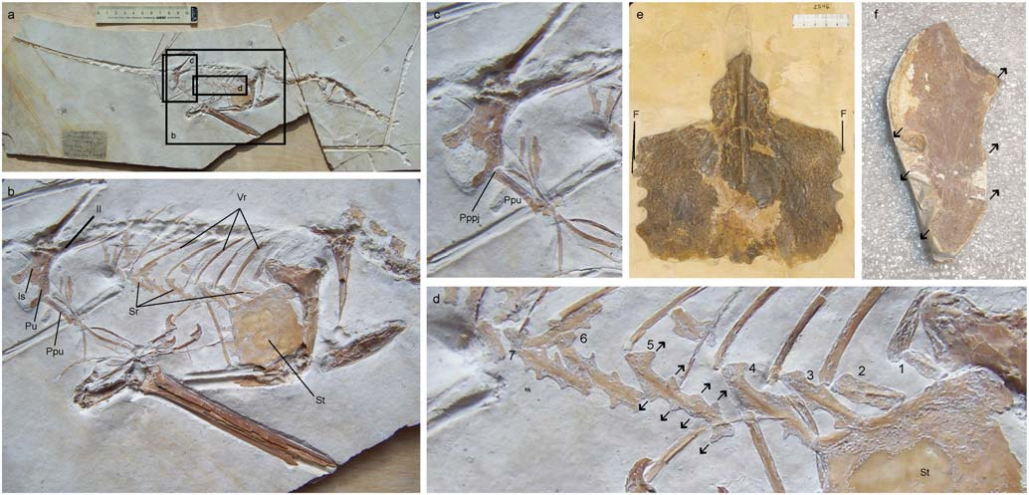
Thoracic and pelvic anatomy of the basal pterosaur *Rhamphorhynchus* (a–d) and the pterodactyloid *Pteranodon* (e, f). a, *Rhamphorhynchus muensteri* (MB-R. 3633.1-2) showing the location of magnified sections b through d. b, Trunk, showing the location of thoracic and pelvic bones. c, Pelvis, right lateral view, showing the location of the pubis-prepubis joint and the medial prepubic prong. d, Sternal ribs 1 through 7, illustrating the ordered arrangement of sternocostapophyses that act as levers for the intercostal muscles (black arrows). e, Sternum of *Pteranodon* (YPM 2546), showing fragments of the distal sternal ribs articulating with the costal facets of the sternum. f, Complete sternal rib of *Pteranodon* (YPM 1175), showing the erose sternal rib margins but ordered distribution of the sternocostapophyses. Scale (a, e) is in centimeters. Abbreviations: F: fragments of distal sternal ribs, Il: ilium, Is: ischium, Pppj: pubic-prepubic joint, Ppu: prepubis, Pu: pubis, Sr: sternal ribs, St: sternum, Vr: vertebral ribs, 1–7, sternal ribs one through seven. Division of Vertebrate Paleontology, YPM 2546 and YPM 1175 (c) 2005 Peabody Museum of Natural History, Yale University, New Haven, Connecticut, USA. All rights reserved.

The ribcage of pterosaurs, including those of the earliest known forms such as the Late Triassic *Eudimorphodon ranzii* [28], consists of a large ossified sternum and distinct vertebral and sternal ribs (Fig. 2a–b, d–f). Intermediate ribs, present in basal lepidosaurs and extant crocodylians [21], are absent in pterosaurs, signalling a reduction of degrees of freedom of movement of the thorax over the basal amniote and archosaur conditions. Cineradiographic investigations of the skeletal kinematics of breathing in the American alligator, *Alligator mississippiensis*, confirm the significance of an additional costal segment for thoracic mobility (Video S1, S2, Table S1), when compared to the bipartite ribcage of birds (Video S3) [26], [27].

**Figure 3:**
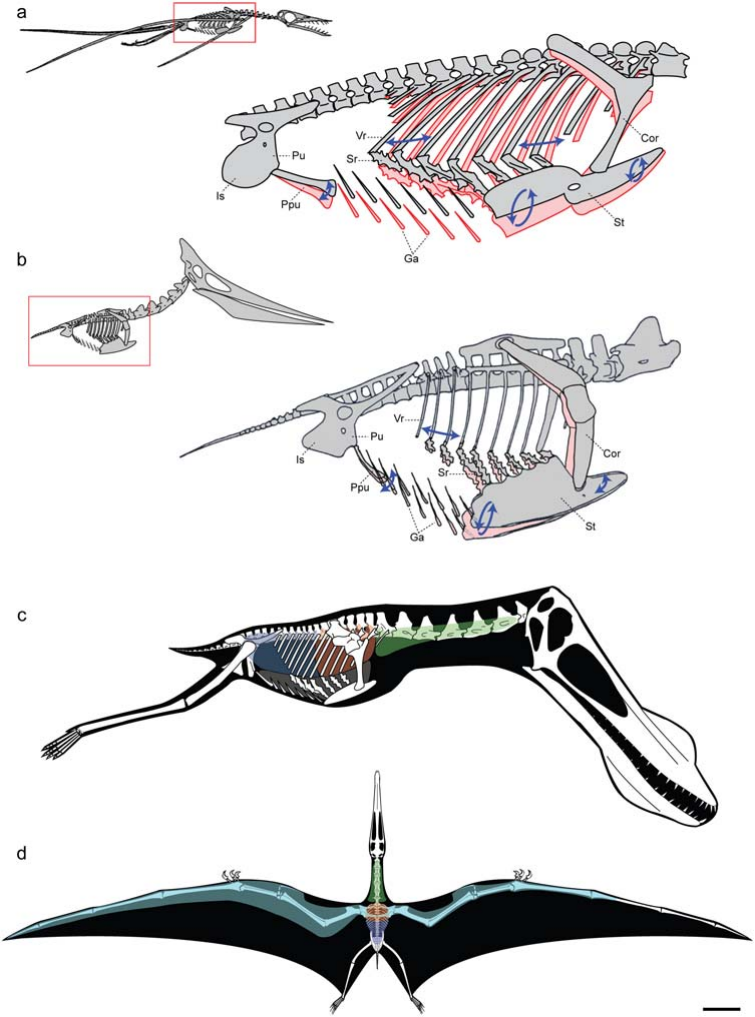
Models of ventilatory kinematics and the pulmonary air sac system of pterosaurs. a, Model of ventilatory kinematics in *Rhamphorhynchus*. Thoracic movement induced by the ventral intercostal musculature results in forward and outward displacement of the distal vertebral and proximal sternal ribs, and ventral displacement of the sternum, upon inspiration (blue arrows and pink outline). In addition, ventral expansion of the abdomen is induced through caudoventral rotation of the prepubis. Ranges of skeletal movement were modelled after those observed *in vivo* in the avian thorax and the crocodylian pelvis [26], [27]. *Rhamphorhynchus* modified from Wellnhofer [48]. b, Model of ventilatory kinematics in *Pteranodon* wherein the fused anterior vertebral ribs and articulation of the scapulocoracoid with the supraneural plate and anterior sternum limit movement of the anterior sternum, which cannot undergo elliptical rotation. However, the posterior vertebral ribs, sternal ribs, sternum, and prepubis are still capable of anterodorsal-posteroventral excursions facilitating volumetric increases and decreases of the thorax during inspiration-expiration. *Pteranodon* modified from Bennett [29]. c, d, reconstruction of pulmonary air sac system in the Lower Cretaceous ornithocheirid *Anhanguera santanae* (AMNH 22555). c, Lateral view showing the inferred position of the lungs (orange), cervical (green) and abdominal air sacs (blue), as predicted on the basis of postcranial skeletal pneumaticity. Thoracic air sacs (shown in grey) are also likely to have been present, but generally do not leave a distinct osteological trace. Humerus and more distal forelimb not shown. d, Dorsal view illustrating the inferred position of subcutaneous diverticular networks (light blue) distally along the wing. The right side depicts a conservative estimate for the size of the airsac network, limiting it to the pre-axial margin of the wing based solely on the presence of pneumatic foramina in closely positioned wing bones. The left side depicts the likely maximal size of an inferred diverticular network, accounting for its inclusion between the dorsal and ventral layers of the wing membrane. Scale = 10 cm. Skeletal reconstruction in c, d modified from Wellnhofer [49]. Abbreviations: as in figure 2, and: Cor: coracoid portion of scapulocoracoid, Ga: gastralia.

The morphology of the trunk of pterosaurs differs from previous descriptions in several aspects that are crucial to lung ventilation and respiratory efficiency. Contrary to earlier reports [1], [7], [29], [30], pterosaur sternal ribs are not of uniform length and posterior elements commonly exhibit a two-fold or greater increase in length (Figs. 2, S1; Table S2). Consequently, and unlike recent reconstructions of pterosaurs which tend to show a horizontal or even posterodorsally sloping sternum, the posterior margin of the pterosaur sternum sloped posteroventrally, similar to birds[21]. As a result, the pterosaur trunk would have been deepest in the posterior sternal region and, due to the longer moment arm of posterior sternal ribs, this region would have undergone the greatest amount of displacement during lung ventilation (Fig. 3a, b).

The sternal ribs of well preserved examples of *Rhamphorhynchus* and *Pteranodon* bear elaborate dorsal and ventral processes that we term sternocostapophyses (Figs. 2d,f, S1c–e). These projections likely functioned as levers that increased the moment arm for the intercostal muscles, conferring an enhanced capacity for moving the sternal ribs during lung ventilation. Sternocostapophyses are analogous in function to the uncinate processes of birds and maniraptoran theropods [31]–[34]. However, the mechanical advantage (leverage) provided by the sternocostapophyses likely differed from that conferred by the uncinate processes of maniraptoran theropods and birds. The sternocostapophyses are located on the sternal ribs rather than the vertebral ribs and, generally, there are multiple sternocostapophyseal projections per sternal rib, rather than a singular (uncinate) process as found in birds. Similar to the uncinate processes of extant birds, the leverage provided by the sternocostapophyseal projections of pterosaurs likely lowered the work of breathing of the intercostal musculature, and resulted in costal and sternal displacement. However, in pterosaurs, the greatest mechanical advantage would have been provided in the ventral rather than dorsal thoracic region.

**Figure 4:**
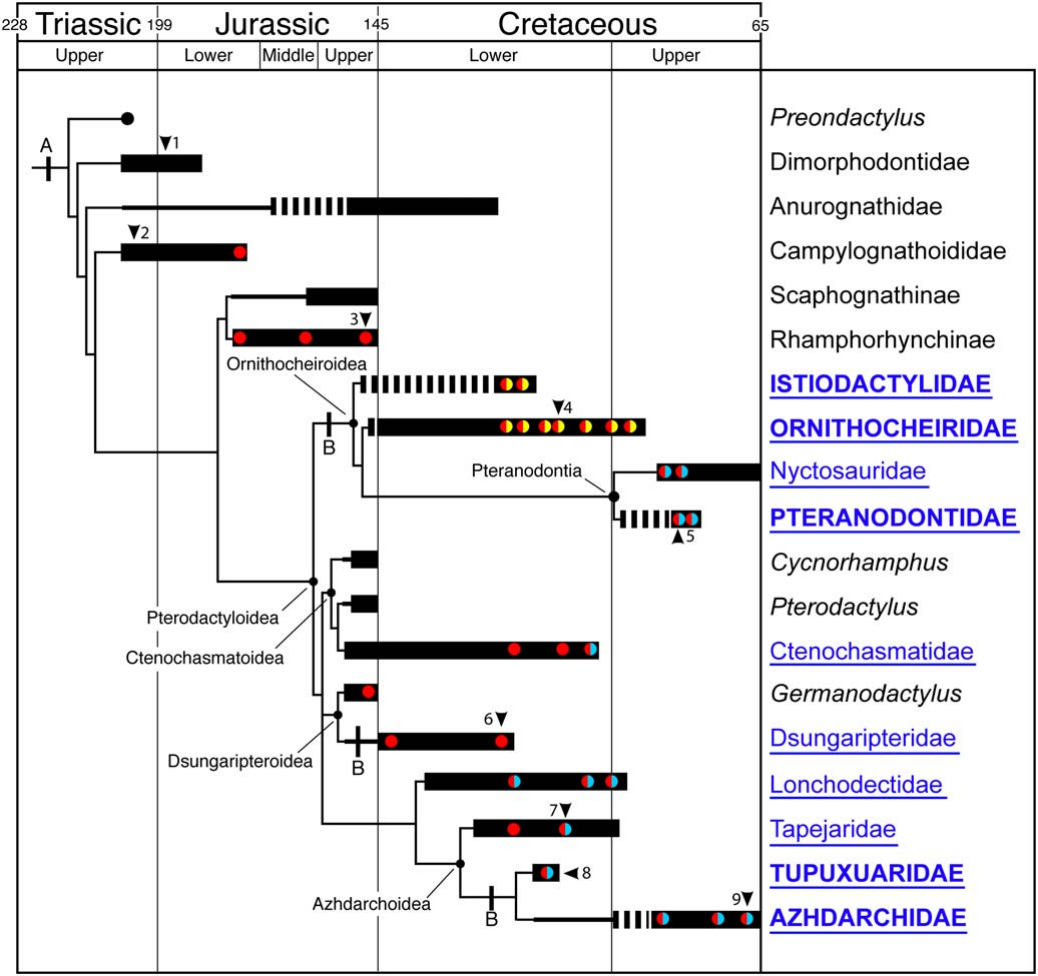
The evolution of the respiratory apparatus in pterosaurs. Tree based on Unwin (2003, 2004), stratigraphic data correct to 2008 (Unwin, unpublished data) and the chronology of Gradstein *et al.* (2004)[50]. Black bars indicate known stratigraphic ranges of the main pterosaur clades, listed at right. Dashed section of bars denotes range extension based on an unverified record. Thick black lines signify a range extension inferred from phylogenetic relationships. Color-filled circles represent occurrences of pneumatization with the following distributions: red = vertebral column; yellow = postaxial pathway in the forelimb; blue = preaxial pathway in the forelimb and in some cases (lonchodectids, *Tupuxuara*, azhdarchids) a limited presence in the hind limb. Clades in which one or more species reached a wingspan of more than 2.5 metres are shown in underlined dark blue text, and more than 5.0 metres, in caps. A, Basic pterosaurian breathing pump (sternum, vertebral and sternal ribs, gastralia and prepubes): B, notarium. Taxa referred to in the text: 1, *Dimorphodon*; 2, *Eudimorphodon*; 3, *Rhamphorhynchus*; 4, *Anhanguera*; 5, *Pteranodon*; 6, *Dsungaripterus*; 7, *Tapejara*; 8, *Tupuxuara*; 9, *Quetzalcoatlus*.

Fusion of vertebral ribs to dorsal vertebrae, and of these vertebrae to one another to form a notarium [1], occurred in many (possibly all) large pterodactyloids (e.g. *Pteranodon*, *Dsungaripterus*, *Tupuxuara* (Fig. 4)), and likely reflects a response to the structural demands placed on this region by stresses transmitted through the body during flight [29], [30]. This rendered the dorsal portion of the thorax immobile, but did not completely restrict thoracic movement as has been suggested [9], [10] (Fig. 3b). Importantly, the presence of elaborate sternocostapophyses in *Rhamphorhynchus* (Fig. 2b,d) demonstrates that the emphasis on ventral sternal displacement in aspiration breathing predated the development of a notarium in large pterodactyloids (Fig. 4). Consequently, movements initiated by sternal rib musculature were capable of generating significant dorsoventral excursions of the sternum in all pterosaurs, and provide a solution to the paradox of pterodactyloid thoracic immobility [9], [10] (Fig. 3a, b).

The gastralia and prepubes also contributed to lung ventilation. During inspiration the metameric rows of gastralia likely stiffened the ventral body wall, helping to prevent it from moving inwards and encroaching on pulmonary air space [35]. Concurrently, the prepubes, which articulated with the puboischial plate via a cranioventral joint (Fig. 2c), were rotated caudoventrally through contraction of pelvic muscles, increasing trunk volume in a manner analogous to that performed by the crocodylomorph pubis [8], [12], [22]. The structural integrity conferred on the abdominal wall by the gastralia likely further facilitated the dorsal displacement of the abdominal wall during expiration, and ventral displacement of the abdominal wall upon inspiration, as observed in extant alligators [26]. Due to the absence of an imbricating metameric midline articulation of the gastralia, lateral expansion of the ventral abdominal wall through gastralial protraction, as hypothesized for theropods [23], did not occur.

The skeletal breathing pump of pterosaurs, including the vertebral and sternal ribs, sternum, gastralia and prepubes, likely formed a highly integrated functional complex. The persistence of the basic components of this system in all pterosaur clades suggests that our inferences related to ventilatory mechanics, and primarily based upon *Rhamphorhynchus* and *Pteranodon*, can be safely assumed to have generally applied to the group.

The aspiration pump of pterosaurs maximised trunk expansion in the ventrocaudal region, while at the same time limiting the degrees of freedom of movement of the trunk in other directions. This provided greater control over the location, amount and timing of trunk expansion, thereby enabling precisely-timed localized generation of pressure gradients within the pulmonary system, a trait that is also present in living birds where it is of paramount importance for the generation of air flow patterns in the lungs [27], [36].

### Structure and function of the pulmonary apparatus

Along with living birds and saurischian dinosaurs, pterosaurs are the only vertebrates that exhibit unambiguous evidence for pneumatization of the postcranial skeleton by pulmonary air sacs [18]–[20], [24], [37], [38], a process in which respiratory epithelium invades portions of the postcranial skeleton leaving distinct openings and excavations in the bones [20], [39]. An analogous system of postcranial skeletal pneumatization is known in a species of osteoglossomorph fish, *Pantodon*, although the gas bladder is the pneumatizing system [40]. Pneumaticity of the vertebral column is widespread in pterosaurs, but variable from group to group within Pterosauria (Fig. 1,4). Where present in basal taxa, pneumaticity appears to be restricted to the dorsal vertebrae and vertebrae at the cervicodorsal transition. This is variably expanded into the cervical and sacral series in pterodactyloids and some relatively derived basal forms such as *Rhamphorhynchus* [41], and extends into the sacral vertebral series and into the ilium and pubis in *Anhanguera santanae* (Figs. 1 a–f, 4, S2; Text S1).

Until recently, the relationship between specific avian air sacs and the regions they pneumatize remained ambiguous, but, now, strict correlations between specific air sacs and the skeletal elements pneumatized exclusively by these air sacs in living birds have been established [18], [19]. The exclusive correlation between, for example, the abdominal air sacs and pneumaticity of the sacral vertebrae [18], [19] and pelvic bones [18], [24] has permitted inferences regarding pulmonary anatomy in extinct theropods based on skeletal pneumaticity patterns [18], [19], [24]. Patterns of pneumaticity of the vertebral column as well as other skeletal elements (Figs. 1, S2, Table S4) suggest, by analogy with birds, that pterosaurs possessed a heterogeneously partitioned pulmonary system, composed of both exchange (lung) and non-exchange (air sac) regions, with distinct anterior (cervical) and posterior (abdominal) components (Figs. 3c,d, S3).

The presence of distinct highly compliant air sac regions, both anterior to, and posterior to the gas exchange region of the pulmonary system (Fig. 3c), is indicative of a flow-through model for the pterosaurian lung, analogous to that recently proposed for theropods [19], [24]. We would like to stress, as we have in previous studies [18], [19], that a flow-through model does not specify the specific type of intrapulmonary airflow pattern that is generated during lung ventilation, which may have been either bidirectional or unidirectional. A bidirectional air flow regime likely predated unidirectional air flow in the evolution of extremely heterogeneous sauropsid respiratory systems, such as for instance the avian pulmonary apparatus. The potential for double aeration, and thus two episodes of gas exchange per breath, in the intermediately-positioned respiratory epithelium, by air that is drawn into the posterior air sac region of the lung, already offers a theoretical increase in respiratory efficiency over the basal sac like or multi-chambered sauropsid lung, or the terminal alveolar pulmonary design of mammals. Notably, such a flow plan is mirrored in the avian neopulmo.

### Appendicular pneumaticity and aerial gigantism

Pneumatization of the appendicular skeleton appears to be highly restricted or absent in basal pterosaurs, ctenochasmatoids and dsungaripteroids (Fig. 4). By contrast, pneumatization of the limb girdles and limb elements is widespread in ornithocheiroids such as *Pteranodon* and *Anhanguera*; the latter group exhibits pneumatic invasion of virtually the entire axial and forelimb skeleton, including distal components of the carpus and manus (Fig. 1c–d, 4). Azhdarchoids (e.g. *Tupuxuara*, *Quetzalcoatlus*) exhibit pneumaticity of the same limb elements, but pneumatic foramina are often located in different positions, suggesting an independent origin and evolution of appendicular pneumaticity in these clades. There is a strong correlation between pneumaticity and size. Pneumaticity is generally absent in small pterosaurs, or confined to the vertebral column, but is almost always present in individuals with wingspans in excess of 2.5 metres and seemingly universal in all taxa with wingspans of 5 metres or more (Fig. 4). This suggests that density reduction via the replacement of bone and bone marrow by air-filled pneumatic diverticula likely played a critical role in circumventing limits imposed by allometric increases in body mass, enabling the evolution of large and even giant size in several clades.

In birds, pneumaticity of forelimb elements distal to the elbow is restricted to large-bodied forms such as pelicans, vultures and bustards (Table S3). In these birds an extensive subcutaneous diverticular network, originating from the clavicular air sac, is responsible for pneumatization of skeletal elements distant from the main pulmonary system [20]. The occurrence of pneumatic foramina in distal limb elements of ornithocheiroids and azhdarchoids, and of a layer of spongy subdermal tissue in an exceptionally well-preserved fragment of wing membrane of an azhdarchoid pterosaur [16], [42], together suggest that a subcutaneous air sac system was present in at least some pterodactyloids. The primary role of such a system is likely to have been density reduction, as in birds [43], but it may have had other advantages. Differential inflation of subcutaneous air sacs along the wing membrane could have altered the mechanical properties (e.g., relative stiffness) of flight control surfaces in large-bodied pterodactyloids (Fig. 3d). In addition, this system may have assisted with thermoregulation [16], and could have also served as an intra- or interspecific signalling device during display behavior, similar to some living birds [44]. Thus, the presence of a subcutaneous air sac system likely played an important role in the functional and ecomorphological diversification of pterodactyloid pterosaurs.

### Conclusions

The evidence for a lung-air sac system and a precisely controlled skeletal breathing pump supports a flow-through pulmonary ventilation model in pterosaurs, analogous to that of birds. The relatively high efficiency of flow-through ventilation was likely one of the key developments in pterosaur evolution, providing them with the respiratory and metabolic potential for active flapping flight and colonization of the Late Triassic skies. This interpretation is consistent with other lines of evidence supporting relatively high metabolic rates in pterosaurs, including the filamentous nature of the integument [e.g. 16], [45], [46], a flight performance comparable to that of extant birds and bats [1], [3], [4], [6], [7], [16], [17] and relatively large brain size [47]. The expansion of a subcutaneous air sac system in the forelimb facilitated the evolution of gigantism in several derived pterodactyloid groups and resulted in the emergence of the largest flying vertebrates that ever existed.

## Methods

### μCT Imaging and Visualization

Pterosaurian skeletal elements were scanned on both clinical and micro-computed tomography (CT) scanners. Large specimen (>127.5 mm) computed tomography was conducted on a GE Lightspeed 16 CT scanner housed at the Stony Brook University Hospital. Smaller specimens (e.g., syncarpals) were scanned on a GE eXplore Locus *in-vivo* micro-CT scanner at the Ohio University microCT Facility.

Elements scanned on the GE Lightspeed 16 were acquired at 120 kVp, 100 mA, and a slice thickness of 0.950 mm, whereas those scanned on the GE eXplore Locus were acquired at 85 kVp, 400 mA, and a slice thickness of 0.045 mm.

VFF (GE output) and DICOM files were compiled into three-dimensional reconstructions on a Dell Precision 670 3.8 GHz Xeon with 4 GB of memory, and an nVidia Quadro FX 4400 512 MB graphics card. Visualizations were obtained using AMIRA 4.1 Advanced Graphics Package.

### Cineradiographic analysis of skeletal kinematics during lung ventilation

Movements of the trunk skeleton in the American alligator (*Alligator mississippiensis*) and birds (*Dromaius novaehollandiae*, *Numida meleagris*, and *Nothoprocta perdicaria*) were filmed using high-speed cineradiography (X-ray filming). Cineradiography was undertaken with a Siemens system employing 16 mm Kodak Eastman Plus-X reversal film and Mini Digital Video. Still images were recorded on Kodak Industrex M-2 film. Kinematic data were recorded at 220 mA, 38 kV, 100 frames per second (fps) using a Photosonics series 2000 high speed cine camera. Digital video was recorded with a Sony DCR VX 1000 camera at 60 fps and 1/250 shutter speed at 220 mA and 50–90 kV. Skeletal movements were recorded in lateral and dorsoventral projection, and were analyzed using Adobe Premiere, Photoshop, NIH Image, and Macromedia Flash. All animal experiments were conducted in accordance with State and Institutional guidelines. *In vivo* movements were correlated with joint anatomy and structure in extinct archosaurs.

### Institutional Abbreviations

**AMNH**, American Museum of Natural History, New York (USA)

**BMNH**, Natural History Museum, London (UK)

**BSPG**, Bayerische Staatssammlung für Paläontologie und Geologie, Munich (Germany)

**CM**, Carnegie Museum, Pittsburgh (USA)

**CAMSM**, Sedgwick Museum, Cambridge (UK)

**IMCF**, Iwaki Museum of Coal and Fossils, Iwaki (Japan)

**MB**, Museum für Naturkunde der Humboldt Universität, Berlin (Germany)

**MGUH**, Geological Museum, Copenhagen (Denmark)

**SMNS**, Staatliches Museum für Naturkunde Stuttgart (Germany)

**TMP**, Royal Tyrrell Museum of Palaeontology, Alberta (Canada)

**TSNIGR**, Central Geological Research Museum, Saint Petersburg (Russia)

**USNM**, United States National Museum, Smithsonian Institution, Washington D.C. (USA)

**YPM**, Yale Peabody Museum of Natural History, New Haven (USA)

## Supporting Information

Figure S1 Margins of the sternal ribs of Pteranodon and Rhamphorhynchus. 1 a, Oblique view of the margin of the small bone fragments preserved in articulation with the sternum of YPM 2546, arrow marks the internal trabeculae and the lack of cortical bone around the proximal margin, indicating the fragmentary nature of the “sternal ribs” associated with YPM 2546. Scale = 1 mm. 1 b, abraded bone fragment (arrow) associated with Pteranodon sternum YPM 2692 lacking a well-defined cortical surface, which therefore also cannot represent a complete sternal rib. 1 c, Elongate sternal rib (arrow) with sternocostapophyses, Pteranodon YPM 2626. 1d, Elongate sternal ribs (arrows) with sternocostapophyses, Pteranodon UALVP 24238. Scale = 25 mm. 1e, Elongate sternal ribs (arrows) with sternocostapophyses in Rhamphorhynchus JME SOS 2819, previously described as fish bone gut content [51]. Scale = 1 cm. In addition to JME SOS 2819 and MB-R. 3633.1-2, similar erose sternal ribs are present in USNM 2420 and can be seen on a photograph published in (Gross, 1937) [52]. Division of Vertebrate Paleontology, YPM 2546, YPM 2626, and YPM 2692 (c) 2005 Peabody Museum of Natural History, Yale University, New Haven, Connecticut, USA. All rights reserved.
(5.78 MB TIF)

Figure S2 Pneumatic features preserved in the postcranial axial skeleton and the appendicular skeleton of Anhanguera santanae (AMNH 22555). 2a, sixth cervical vertebra, right lateral view; 2b, fourth cervical vertebra, cranial view; 2c, ultimate cervical (*) and cranial dorsal (thoracic) vertebral series, left dorsolateral view. 2d, proximal left humerus, anterior view (inset showing close-up of pneumatic foramen); 2e, left proximal syncarpal, distal view; 2f, left distal syncarpal, proximal view. Black arrows indicate pneumatic openings. Scale equals 1 cm.
(3.66 MB TIF)

Figure S3 Micro-computed tomographic (CT) scan of a Great skua (Catharacta skua-CM 11606). a, b, Posterior cervical vertebra in oblique craniolateral (a) and cutaway oblique craniolateral (b) views, showing the high level of pneumatic excavation, similar to Anhanguera. Abbreviations similar to text Figure 1. Vertebral height of specimen = 15 mm.
(3.74 MB TIF)

Table S1 Excursions of the vertebral and intermediate ribs in the American alligator, Alligator mississippiensis (Table after Claessens, In Press26). Average anterior and lateral displacement of the distal vertebral rib and the distal intermediate rib upon inspiration. Angle with longitudinal body axis: α. Relative distance of displacement, measured as a function of the furthest displaced rib within the thorax: λ, where λ = (displacement rib/ maximally displaced rib within thorax)×100.
(0.04 MB DOC)

Table S2 Increase in length of the longest (posterior) sternal ribs as a function of the shortest (anterior) sternal ribs in three pterosaur taxa. Values indicated by an asterisk are estimated due to loss of material or obstruction of sternal ribs by matrix or other skeletal elements. In extant birds, relative increase in sternal rib length generally exceeds 100% (n = 60).
(0.03 MB DOC)

Table S3 List of large-bodied extant birds exhibiting distal forelimb pneumaticity. In all cases distal forelimb pneumaticity is associated with an extensive subcutaneous air sac system that passes distally down the wings.
(0.03 MB DOC)

Table S4 Key pterosaur specimens exhibiting pneumatic features. Pneumaticity was defined as the presence of pneumatic foramina in the bony cortex, as opposed to the presence of “pneumatic” fossae, which may be the product of diagenetic effects and various biological processes other than pneumatic diverticulae induced bone remodeling [18]:
(0.10 MB DOC)

Text S1 Supplementary Text S1 and Additional References
(0.04 MB DOC)

Video S1 Cineradiographic (X-ray film) clip of a 1.0 kg female American alligator (Alligator mississippiensis), demonstrating the role of the intermediate rib in thoracic narrowing during expiration, and thoracic widening during inspiration. Experimental subject in lateral projection at 70 kV and 220 mA, X-ray positive. Head is toward right side of image. (see appended Quicktime file).
(6.92 MB MOV)

Video S2 Cineradiographic (X-ray film) clip of a 1.0 kg female American alligator (Alligator mississippiensis), demonstrating the role of the intermediate rib in thoracic narrowing during expiration, and thoracic widening during inspiration. Experimental subject in dorsoventral projection at 70 kV and 220 mA, X-ray positive. Head is toward bottom of image. (see appended Quicktime file).
(6.33 MB MOV)

Video S3 Cineradiographic (X-ray film) clip of a 2.1 kg helmeted guinea fowl (Numida meleagris), demonstrating the uniformity of thoracic widening in absence of an intermediate rib. Experimental subject in dorsoventral projection at 70 kV and 220 mA, X-ray positive. Head is toward bottom left of image. (see appended Quicktime file).
(3.94 MB MOV)
