# Supplementary figures and images for: Respiratory Evolution Facilitated the Origin of Pterosaur Flight and Aerial Gigantism
Source: PLoS One. 2009 Feb 18;4(2):e4497. doi: 10.1371/journal.pone.0004497 (PMC2637988; doi:10.1371/journal.pone.0004497)

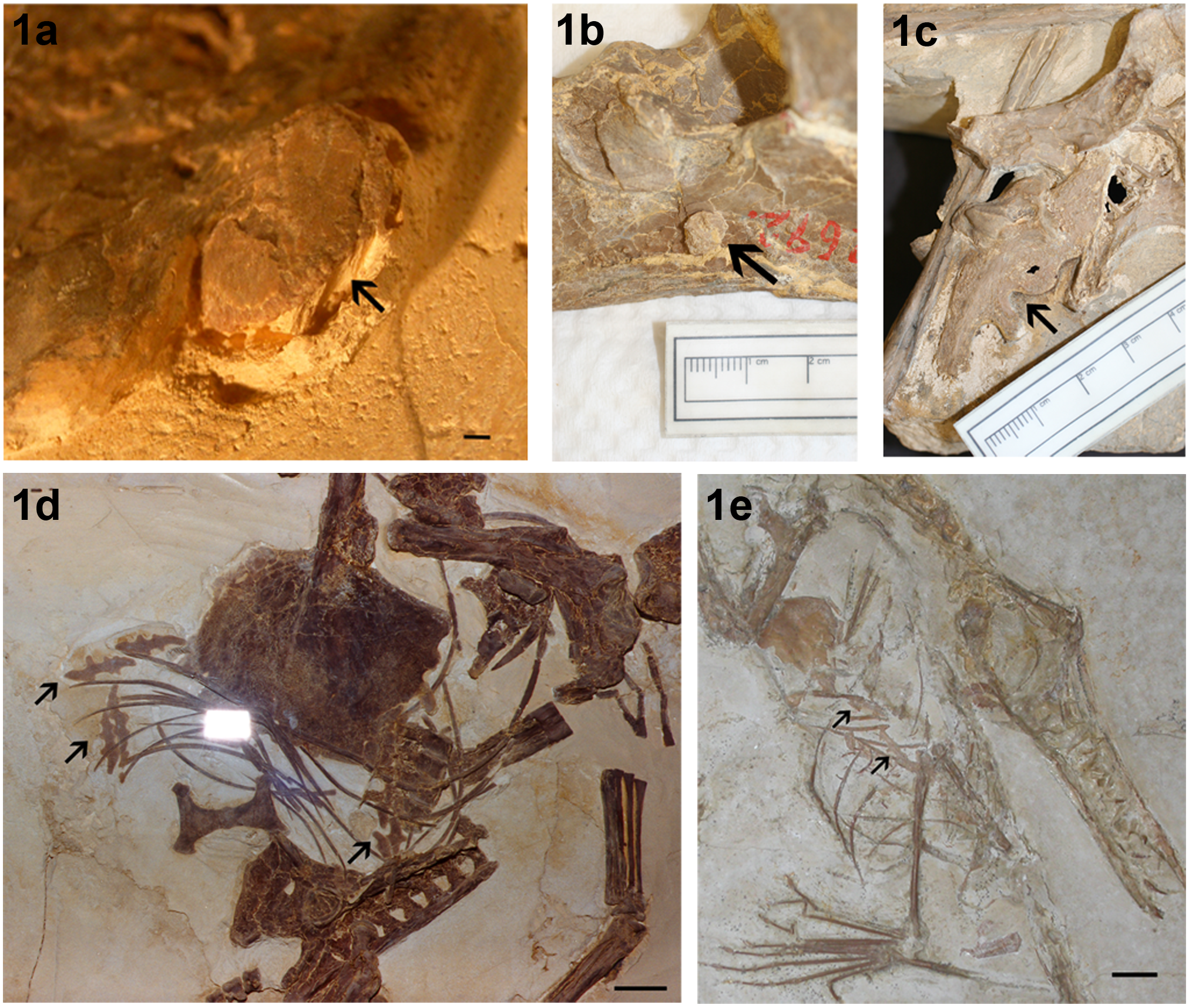

Supplement: Figure S1 — Margins of the sternal ribs of Pteranodon and Rhamphorhynchus. 1 a, Oblique view of the margin of the small bone fragments preserved in articulation with the sternum of YPM 2546, arrow marks the internal trabeculae and the lack of cortical bone around the proximal margin, indicating the fragmentary nature of the “sternal ribs” associated with YPM 2546. Scale = 1 mm. 1 b, abraded bone fragment (arrow) associated with Pteranodon sternum YPM 2692 lacking a well-defined cortical surface, which therefore also cannot represent a complete sternal rib. 1 c, Elongate sternal rib (arrow) with sternocostapophyses, Pteranodon YPM 2626. 1d, Elongate sternal ribs (arrows) with sternocostapophyses, Pteranodon UALVP 24238. Scale = 25 mm. 1e, Elongate sternal ribs (arrows) with sternocostapophyses in Rhamphorhynchus JME SOS 2819, previously described as fish bone gut content [51]. Scale = 1 cm. In addition to JME SOS 2819 and MB-R. 3633.1-2, similar erose sternal ribs are present in USNM 2420 and can be seen on a photograph published in (Gross, 1937) [52]. Division of Vertebrate Paleontology, YPM 2546, YPM 2626, and YPM 2692 (c) 2005 Peabody Museum of Natural History, Yale University, New Haven, Connecticut, USA. All rights reserved. (5.78 MB TIF) [file pone.0004497.s001.tif]

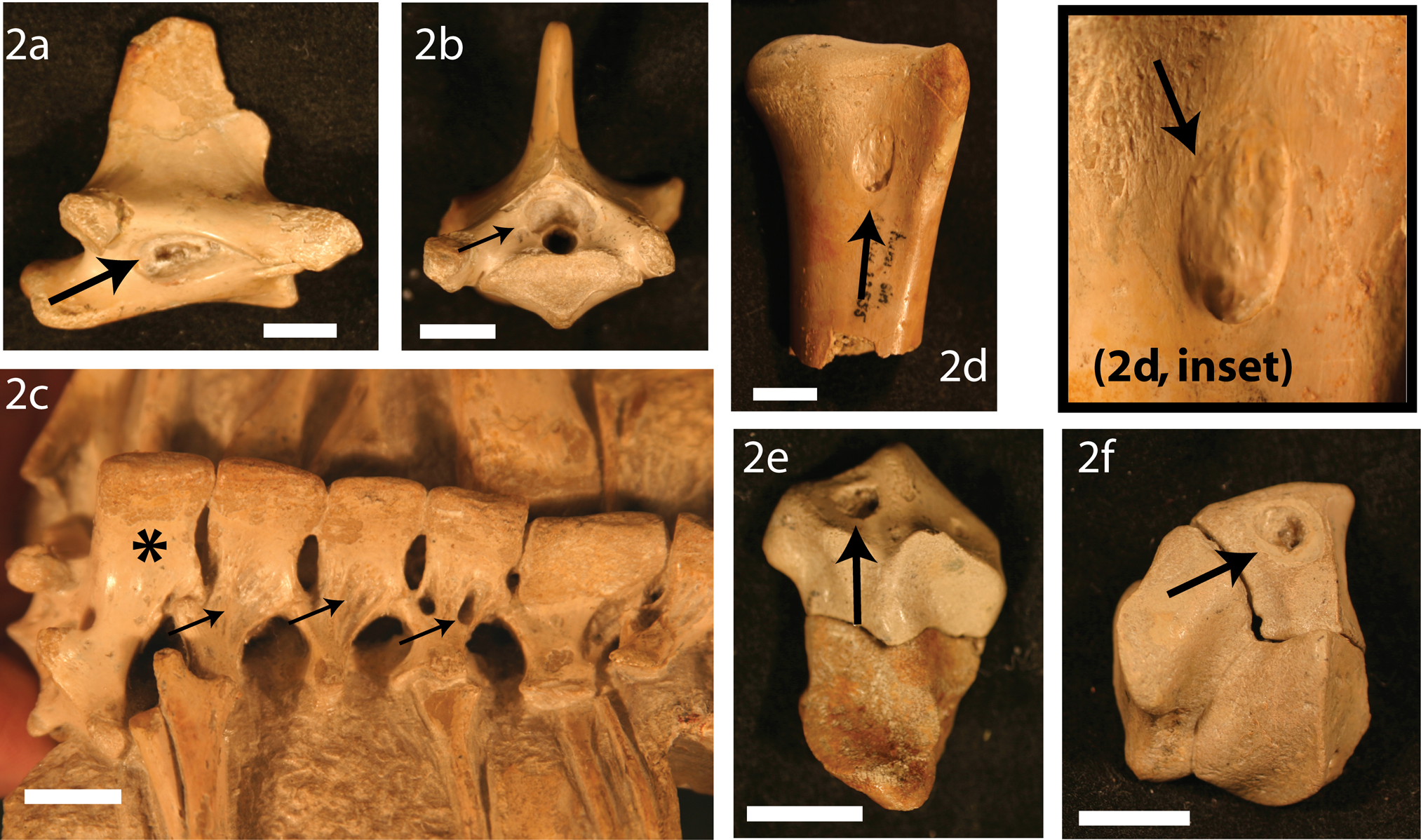

Supplement: Figure S2 — Pneumatic features preserved in the postcranial axial skeleton and the appendicular skeleton of Anhanguera santanae (AMNH 22555). 2a, sixth cervical vertebra, right lateral view; 2b, fourth cervical vertebra, cranial view; 2c, ultimate cervical (*) and cranial dorsal (thoracic) vertebral series, left dorsolateral view. 2d, proximal left humerus, anterior view (inset showing close-up of pneumatic foramen); 2e, left proximal syncarpal, distal view; 2f, left distal syncarpal, proximal view. Black arrows indicate pneumatic openings. Scale equals 1 cm. (3.66 MB TIF) [file pone.0004497.s002.tif]

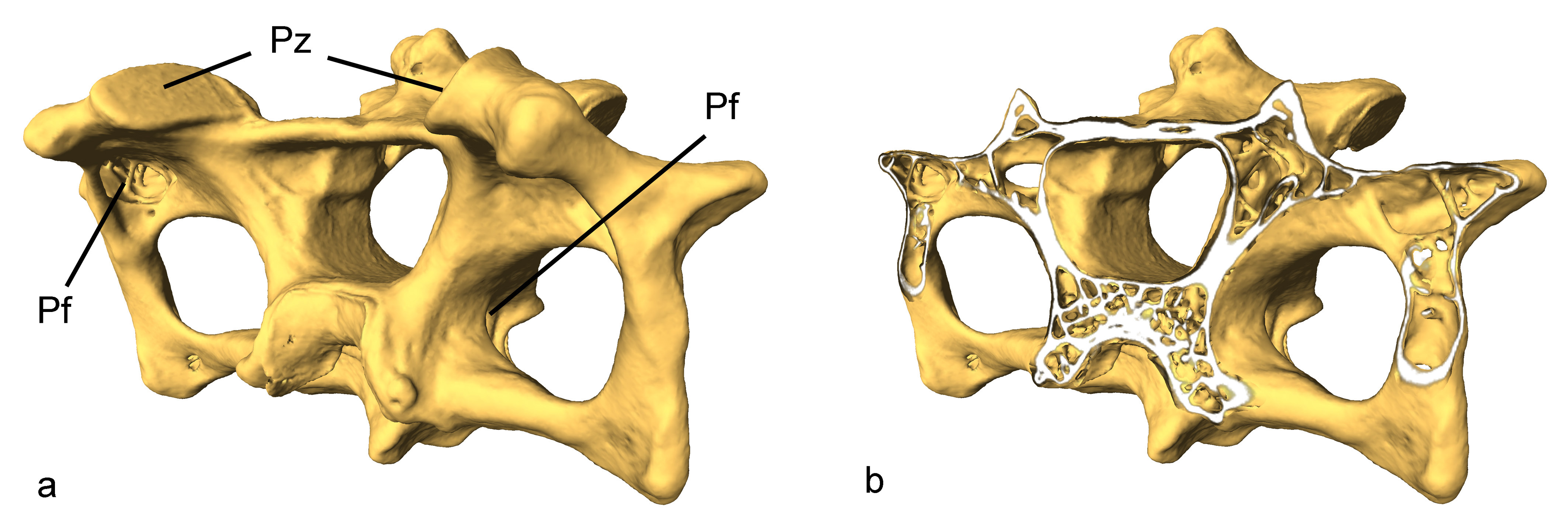

Supplement: Figure S3 — Micro-computed tomographic (CT) scan of a Great skua (Catharacta skua-CM 11606). a, b, Posterior cervical vertebra in oblique craniolateral (a) and cutaway oblique craniolateral (b) views, showing the high level of pneumatic excavation, similar to Anhanguera. Abbreviations similar to text Figure 1. Vertebral height of specimen = 15 mm. (3.74 MB TIF) [file pone.0004497.s003.tif]
