# Supplementary material for: Respiratory Evolution Facilitated the Origin of Pterosaur Flight and Aerial Gigantism
Source: PLoS One. 2009 Feb 18;4(2):e4497. doi: 10.1371/journal.pone.0004497 (PMC2637988; doi:10.1371/journal.pone.0004497)
Supplement: Table S1 — Excursions of the vertebral and intermediate ribs in the American alligator, Alligator mississippiensis (Table after Claessens, In Press26). Average anterior and lateral displacement of the distal vertebral rib and the distal intermediate rib upon inspiration. Angle with longitudinal body axis: α. Relative distance of displacement, measured as a function of the furthest displaced rib within the thorax: λ, where λ = (displacement rib/ maximally displaced rib within thorax)×100. (0.04 MB DOC) [file pone.0004497.s004.doc]

| **Alligator** |  | **Anterior and Lateral Rib Displacement** | | | | | | **Sample Size (N)** |
| --- | --- | --- | --- | --- | --- | --- | --- | --- |
|  | Rib | **1** | | **5** | | **8** | |  |
|  |  | **α** | **λ** | **α** | **λ** | **α** | **λ** |  |
| **1** | Vertebral | 21 | 49 | 47 | 100 | 58 | 78 | 5 |
|  | Intermediate | 37 | 25 | 45 | 100 | 63 | 91 | 5 |
| **2** | Vertebral | 18 | 41 | 42 | 100 | 57 | 72 | 5 |
|  | Intermediate | 27 | 27 | 38 | 100 | 54 | 77 | 5 |
| **3** | Vertebral | 28 | 50 | 54 | 95 | 61 | 76 | 5 |
|  | Intermediate | 43 | 32 | 42 | 100 | 56 | 100 | 5 |
