# Supplementary material for: Respiratory Evolution Facilitated the Origin of Pterosaur Flight and Aerial Gigantism
Source: PLoS One. 2009 Feb 18;4(2):e4497. doi: 10.1371/journal.pone.0004497 (PMC2637988; doi:10.1371/journal.pone.0004497)
Supplement: Table S2 — Increase in length of the longest (posterior) sternal ribs as a function of the shortest (anterior) sternal ribs in three pterosaur taxa. Values indicated by an asterisk are estimated due to loss of material or obstruction of sternal ribs by matrix or other skeletal elements. In extant birds, relative increase in sternal rib length generally exceeds 100% (n = 60). (0.03 MB DOC) [file pone.0004497.s005.doc]

| **Taxon** | **Length of shortest sternal rib** | **Length of longest sternal rib** | **Relative increase in sternal rib length** |
| --- | --- | --- | --- |
| Eudimorphodon MCSNB 2888  (From Wild 1978) | 10 mm * | 20 mm * | 100 % |
| Rhamphorhynchus MB-R. 3633.1-2 | 4.7 mm | 11.9 mm | 153 % |
| Pteranodon UALVP 24238 | 23 mm * | 49 mm | 113 % |
