# Supplementary material for: Respiratory Evolution Facilitated the Origin of Pterosaur Flight and Aerial Gigantism
Source: PLoS One. 2009 Feb 18;4(2):e4497. doi: 10.1371/journal.pone.0004497 (PMC2637988; doi:10.1371/journal.pone.0004497)
Supplement: Table S3 — List of large-bodied extant birds exhibiting distal forelimb pneumaticity. In all cases distal forelimb pneumaticity is associated with an extensive subcutaneous air sac system that passes distally down the wings. (0.03 MB DOC) [file pone.0004497.s006.doc]

| Taxon | Common Name | Maximum Body Size |
| --- | --- | --- |
| Anhimidae | Screamers | 5 kg |
| Bucerotids | Hornbills | 5 kg |
| Cathartidae | New World Vultures | 14 kg |
| Ciconiiformes | Storks | 11 kg |
| Otidae | Bustards | 18 kg |
| Pelecaniformes | Pelicans/Gannets | 15 kg |
| Aegypiinae | Old World Vultures | 12.5 kg |
