# Supplementary material for: Respiratory Evolution Facilitated the Origin of Pterosaur Flight and Aerial Gigantism
Source: PLoS One. 2009 Feb 18;4(2):e4497. doi: 10.1371/journal.pone.0004497 (PMC2637988; doi:10.1371/journal.pone.0004497)
Supplement: Table S4 — Key pterosaur specimens exhibiting pneumatic features. Pneumaticity was defined as the presence of pneumatic foramina in the bony cortex, as opposed to the presence of “pneumatic” fossae, which may be the product of diagenetic effects and various biological processes other than pneumatic diverticulae induced bone remodeling [18]: (0.10 MB DOC) [file pone.0004497.s007.doc]

| **Taxon** | **Specimen number** | **Observed Pneumatic Elements** |
| --- | --- | --- |
| **Campylognathoididae** |  |  |
| *Campylognathoides zitteli* | SMNS 51100 | dorsal vertebra |
|  |  |  |
| **Rhamphorhynchinae** |  |  |
| *Dorygnathus banthensis* | SMNS 50702 | anterior dorsal vertebrae |
| *Rhamphorhynchus* | MGUH 1891.738 | cervical and anterior dorsal vertebrae, sternum |
|  |  |  |
| **Istiodactylidae** |  |  |
| *Istiodactylus latidens* | BMNH R176 | humerus |
|  | BMNH 3877 | cervical and dorsal vertebrae, humerus, proximal syncarpal* |
|  |  |  |
| **Ornithocheiridae** |  |  |
| *Ornithocheirus* sp. | SM B54.320 | midcervical vertebra |
|  | SM B54.356 | midcervical vertebral centrum |
|  | SM B54.314 | atlantoaxis |
|  | SM B54.973 | dorsal vertebra |
|  | SM B54.970 | dorsal vertebra |
|  | SM B54.333 | cervical vertebra |
|  | BMNH R558 | humerus |
|  | BMNH R3877 | dorsal vertebrae, ulna |
|  | BMNH R3878 | scapulocoracoid |
|  | BMNH R41637 | phalanx* |
|  | BMNH R41638 | ulna |
|  | BMNH R37954 | carpal* |
|  | BMNH R49003 | phalanx I* |
| *Coloborhynchus* sp. | SM B54.302 | atlantoaxis |
| *Araripesaurus* sp. | BSPG 1982 I 91 | partial skeleton |
|  | BSPG 1982 I 93 | ulna |
| *Araripesaurus (Anhanguera) santanae* | BSPG 1982 I 90 | proximal and distal syncarpals* |
| *Anhanguera santanae* | AMNH 22555 | postatlantal precaudal vertebrae, thoracic ribs, pelvic girdle, ulna, radius, proximal and distal syncarpals |
| *Brasileodactylus araripensis* | BSPG 1991 I 27 | cervical vertebrae, scapulocoracoid |
| *Santanadactylus* sp. | BSPG 1983 I 92 | appendicular elements |
| *Santanadactylus araripensis* (= *Coloborhynchus araripensis*) | BSPG 1982 I 89 | humerus, ulna, carpals* |
| *Santanadactylus brasilensis* | BSPG 1981 I 15-16 | cervical vertebrae |
| *Santanandactylus pricei* (?) | BSPG 1980 I 122 | metacarpal* |
|  |  |  |
| **Pteranodontidae** |  |  |
| *Pteranodon* sp. | USNM 9050 | cervical vertebra |
|  | BMNH R2929 | cervical vertebra |
|  | BMNH R4534 | cervical vertebra |
|  | USNM 13804 | humerus |
|  | USNM 20711 | humerus |
|  | USNM 18266 | metacarpal* |
|  | BMNH R4537 | carpus*, metacarpal* |
|  |  |  |
| **Lonchodectidae** |  |  |
| *Lonchodectes* sp. | BMNH R3694 | humerus, ulna, radius, metacarpal* |
|  |  |  |
| **Tupuxuaridae** |  |  |
| *Tupuxuara longicristatus* | IMCF 1052 | cervical and dorsal vertebrae, humerus, femur |
|  |  |  |
| **Azhdarchidae** |  |  |
| Azhdarchidae | TMP 87.36.16 | wing-metacarpal* |
| *Azhdarcho lancicollis* | TSNIGR 3/11915 | atlas-axis |
|  | TSNIGR 1/11915 | mid-series cervical vertebra |
|  | TSNIGR 5/11915 | mid-series cervical vertebra |
|  | TSNIGR 6/11915 | mid-series cervical vertebra |
|  | TSNIGR 7/11915 | notarium |
|  | TSNIGR 9/11915 | femur |
|  |  |  |
