# Supplementary material for: Respiratory Evolution Facilitated the Origin of Pterosaur Flight and Aerial Gigantism
Source: PLoS One. 2009 Feb 18;4(2):e4497. doi: 10.1371/journal.pone.0004497 (PMC2637988; doi:10.1371/journal.pone.0004497)
Supplement: Text S1 — Supplementary Text S1 and Additional References (0.04 MB DOC) [file pone.0004497.s008.doc]

**Text S1. Pneumaticity profile of *Anhanguera santanae* (AMNH 22555)**

AMNH 22555 preserves a near complete postcranial axial skeleton and numerous components of the appendicular skeleton.

All post-atlantal, precaudal vertebrae of AMNH 22555 exhibit numerous features indicative of pneumatic invasion of bone by pulmonary air sacs and/or diverticula. Moreover, select dorsal (thoracic) ribs also possess pneumatic foramina (at least in the cranially positioned ones that are available for detailed examination).

Pneumatic features range from simple, large foramina on the lateral surface of vertebral centra and neural arches (Suppl. Fig. 2a, b) to complex cortical openings on the dorsal aspect of the dorsal (thoracic) neural arches (Suppl. Fig. 2c). The pelvic girdle, as well as preserved forelimb elements of AMNH 22555, also exhibit pneumatic features, including foramina on the pelvic (e.g., ilium and pubis), antebrachial (ulna and radius) and components of the carpal skeleton (e.g., proximal and distal syncarpals; Suppl. Fig. 2e, f).

# Additional references, supplementary information

51. Wellnhofer P (1975) Die Rhamphorhynchoidea (Pterosauria) der Oberjura-

Plattenkalke Suddeutschlands. Palaeontographica A 148: 132-186.

52. Gross W (1937) Ueber einen neuen *Rhamphorhynchus gemmingi* H. v. M.

des Natur-Museums Senckenberg. Abhandlungen der Senckenbergischen

Naturforschenden Gesellschaft 437: 1-16.
